# Sterol Carrier Protein Inhibition-Based Control of Mosquito Vectors: Current Knowledge and Future Perspectives

**DOI:** 10.1155/2019/7240356

**Published:** 2019-07-10

**Authors:** Hirunika Perera, Tharaka Wijerathna

**Affiliations:** Department of Parasitology, Faculty of Medicine, University of Kelaniya, Colombo, Sri Lanka

## Abstract

Cholesterol is one of the most vital compounds for animals as it is involved in various biological processes and acts as the structural material in the body. However, insects do not have some of the essential enzymes in the cholesterol biosynthesis pathway and this makes them dependent on dietary cholesterol. Thus, the blocking of cholesterol uptake may have detrimental effects on the survival of the insect. Utilizing this character, certain phytochemicals can be used to inhibit mosquito sterol carrier protein-2 (AeSCP-2) activity via competitive binding and proven to have effective insecticidal activities against disease-transmitting mosquitoes and other insect vectors. A range of synthetic compounds, phytochemicals, and synthetic analogs of phytochemicals are found to have AeSCP-2 inhibitory activity. Phytochemicals such as alpha-mangostin can be considered as the most promising group of compounds when considering the minimum environmental impact and availability at a low cost. Once the few limitations such as very low persistence in the environment are addressed successfully, these chemicals may be used as an effective tool for controlling mosquitoes and other disease-transmitting vector populations.

## 1. Introduction

A diverse array of tropical diseases is transmitted to humans through infective insect vectors [1]. These vectors include mosquitoes, sand flies, houseflies, blackflies, tsetse flies, and kissing bugs, which are responsible for the transmission of some of the most virulent diseases in the world [1]. Among these insects, mosquitoes are the most dangerous group. Different species of mosquitoes transmit a range of diseases, namely, malaria, dengue, lymphatic filariasis, dirofilariasis, and Japanese encephalitis [1, 2].

The control of vector-borne diseases depends on the successful control of the vector populations [3]. During the pre-DDT (dichlorodiphenyltrichloroethane) era, main vector control strategies were environmental management and biological control, which included drainage of swamps and other mosquito breeding sites, clearing of vegetation to remove vector resting places and various other traditional methods [4, 5]. With the discovery of DDT as an insecticide, vector control became more effective. However, the uncontrolled use of this chemical caused very serious damage to the ecosystem and animals. Furthermore, the mosquitoes developed resistance to DDT [6–8]. This ruled out the use of a single chemical continuously as the ultimate solution against vector-borne diseases. The resistance was also observed for several other commonly used insecticides [9]. This phenomenon in arthropods pointed out the need for more effective new insecticides to be used in a controlled manner preventing any chance of developing resistance.

Insects do not have some of the essential enzymes in the cholesterol biosynthesis pathway and are incapable of de novo synthesis of cholesterol [10, 11]. This makes them dependent on external sources for cholesterol, an essential compound in their body structures and a precursor for important biocompounds such as molting hormones [12, 13]. Therefore, the inhibition of any vital step of the cholesterol uptake will be fatal to the insect. This can be used as a vector control strategy in the fight against vector-borne diseases. Hence, the current review focuses on discussing available knowledge on the inhibition of cholesterol uptake in mosquitoes with the objective of identifying opportunities and drawbacks of using this method in broadening the tools for vector control.

## 2. Cholesterol as a Vital Compound for Insects

As in most eukaryotic organisms, cholesterol plays an essential role in the stability and architecture of the plasma membranes and acts as a precursor for other metabolites including molting hormones and other signalling molecules which affects the development of insects [14].

Cholesterol is important to maintain the integrity of the membranes and facilitate cell signalling [15–17]. It is an amphipathic molecule with both hydrophilic and hydrophobic domains. Because of this amphipathic nature and the shape of the cholesterol molecule, it binds with the fatty acid chain on the nearest phospholipid molecule making it difficult for some water-soluble molecules to pass through [18]. However, cholesterol also helps in separating phospholipids preventing the crystallization of fatty acid chains by coming together [18, 19]. Thus, cholesterol maintains cellular membrane without becoming too firm or too fluid. Cholesterol also involves in protein sorting and signal transduction between cells by being a part in lipid rafts [20–22], which is very important for the communication between cells preventing the body becoming a group of unrelated cells.

The molt-promoting hormones such as ecdysone and 20-hydroxyecdysone in insects are produced using cholesterol as precursors [14, 23]. These hormones are commonly known as ecdysteroids, and they are essential for the molting process of insects which in turn facilitate the growth and development [24–26]. The juvenile hormone (JH) in insects prevents the secretion of ecdysteroids, and the decline of JH to zero results in the secretion of ecdysteroids which initiates the next molt. Subsequent hormonal actions result in the rise of JH levels, which decrease the ecdysteroid hormone secretion. The ecdysis is triggered by this decline of ecdysteroid levels [26, 27]. In the absence of cholesterol at required levels to maintain these hormone levels, ecdysis or the molting in insects will not take place and may result in growth disruptions, which cause the death of the insect at the larval stage without further development [26, 27].

Cholesterol synthesis takes place within animal and plant cells through a series of enzyme-catalyzed reactions. Biosynthesis of cholesterol uses acetyl-CoA produced in mitochondria through glycolysis or *ß*-oxidation of fatty acids in mitochondria and peroxisome as precursors [28, 29]. The first step is the formation of mevalonate, which is initiated by the condensation of two acetyl-CoA molecules to form acetoacetyl-CoA in the presence of enzyme thiolase. Next, HMG-CoA synthase incorporates another acetyl-CoA molecule to produce HMG-CoA. As the final and the rate-limiting step of mevalonate production, HMG-CoA reductase converts HMG-CoA into mevalonate [30, 31]. Following a series of successive enzyme-catalyzed reactions, farnesyl pyrophosphate is produced. In the presence of squalene synthase, farnesyl pyrophosphate is converted to 30-carbon molecule squalene, which is the precursor for the production of all steroids [31]. Squalene is first converted to lanosterol, and various cholesterols are produced through discrete reactions. These cholesterols are either used as precursors for hormones and structural components of the cells and tissues or stored in fat cells in body tissues such as adipose tissue, liver, and gall bladder [32]. The enzyme, squalene synthase which is essential for the conversion of farnesyl pyrophosphate into squalene in this cholesterol synthesis pathway, and at least two other key enzymes, namely, squalene monooxygenase and lanosterol synthase are absent in insects [10, 11, 33]. This is the reason why insects are incapable of de novo synthesis of cholesterol and thus solely rely on dietary sources to obtain sterols such as phytols, stigmasterol *ß*-sitosterol, and campesterol essential for the growth, development, and reproduction [34].

## 3. Sterol Carrier Protein-Mediated Cholesterol Uptake and Its Inhibition

Cholesterol must be transported from midgut to the site of utilization through membranes and hydrophilic media, such as intercellular fluid, blood, and cellular fluids. This is done only with a way to shield it from the aquaphilic environment. The proteins in the SCP-2 gene family, sterol carrier protein-2 (SCP-2) and sterol carrier protein–-x (SCP-x), have been identified as molecules which assist the organism for the transport of cholesterol through these hydrophilic environments [35, 36].

Unlike vertebrates, insect cells do not transfer cholesterol between the midgut and the fat body through receptor-mediated endocytosis of lipoproteins [37]. Due to its hydrophobic nature, cholesterol may readily diffuse into the outer layer of the cell membrane and proteins such as SCP-2 desorb the molecules from the membrane and deliver them to site of storage or into metabolic pathways [38]. Therefore, any method which reduces the amount of available SCP-2 for cholesterol to bind may reduce the cholesterol uptake in an insect. At the genetic level, overexpression or knockdown of AeSCP-2 gene expression is shown to affect cholesterol uptake [39–41]. The knockdown of AeSCP-2 in larva by the injection of small double-stranded AeSCP-2 RNA resulted in high mortalities in developing adults and reduced egg viability in *Aedes aegypti* [40]. Rearing of larvae in radioactive [3H] cholesterol and measuring the level of [3H] cholesterol in extracted lipids indicated that knockdown of the AeSCP-2 gene resulted in 33% reduction of accumulated cholesterol in pupae [40]. In the presence of a molecule capable of binding with the same active site as cholesterol, the amount of AeSCP-2 available for cholesterol to bind is reduced. This inhibits or significantly reduces the uptake of cholesterol. Many studies have made attempts to discover chemical compounds that inhibit the action of SCP-2 via competitive binding. Some of them are synthetic organic compounds [42], while some of them are phytochemicals [43–45]. Also, there are some synthetic analogs of phytochemicals which have shown to have the ability to inhibit the action of SCP-2. Around 57 organic compounds have been identified as sterol carrier protein inhibitors (SCPIs), out of around 16,000 compounds from chemical libraries [42]. Among these, five major organic compounds have been identified based on the AeSCP-2 inhibitory activity. These were named SCPI-1, SCPI-2, SCPI-3, SCPI-4, and SCPI-5 (Table 1). The affinity to AeSCP-2 has shown promising results where SCPI-5 and SCPI-2 were having a higher affinity than others. This study by Kim et al. [42] revealed that in mosquitoes, the effectiveness of inhibiting the cholesterol uptake varied in the order SCPI-3 > SCPI-2 > SCPI-4 > SCPI-1 > SCPI-5. Both mosquito sterol carrier protein-2 (AeSCP-2) and mosquito sterol carrier protein-x (AeSCP-x) are inhibited, but AeSCP-2 inhibition found to be more effective in making lethal impacts on mosquitoes. Subsequent studies revealed that SCPI-1 and SCPI-2 have broad toxicity in mosquito species such as *Anopheles gambiae*, *Culex quinquefasciatus*, and *Culex pipiens* [36, 46]. Furthermore, it is reported that there is an increase in the toxicity of insecticides such as permethrin when used with AeSCP-2 inhibitors [46]. Moreover, it is also evident that the AeSCP-2-mediated cholesterol uptake pathway is important for dengue viral production in *Aedes* mosquitoes and the inhibition of AeSCP-2 activity results in repressed virus production within mosquito cells in vitro [47]. Curcumin is another compound which is known to have various biological properties including antimicrobial, antioxidant, anti-inflammatory, and anticancer properties [48]. Synthetic analogs of this compound are also known to have AeSCP-2 inhibitory activity [43]. Among these (Table 1), 1,5-bis-(3,4,5-trimethoxy-phenyl)-penta-1,4-dien-3-one is the most effective compounds in the inhibition of AeSCP-2 activity. However, only one of the curcumin analogs, (2E,6E)-2-6-bis(furan-2-ylmethylene) cyclohexanone analyzed here, exhibited larvicidal activity against *Ae. aegypti* larva.

In addition to these compounds tested for larvicidal activity, some phytochemicals have also shown promising results in inhibiting AeSCP-2 (Table 2). The compound named quercetin, isolated from the plant *Saxifraga stolonifera* (creeping saxifrage), indicated to have an AeSCP-2 inhibitory activity [43]. Another compound named *α*-mangostin isolated from plant *Garcinia mangostana* (mangosteen) has given more promising results as it exhibits larvicidal effects against six species of mosquitoes, namely, *Aedes aegypti*, *Anopheles stephensi*, *Anopheles gambiae*, *Culex pipiens pipiens*, *Anopheles quadrimaculatus*, and *Culex quinquefasciatus* [45].

Alpha-mangostin and panthenol isolated from *Garcinia mangostana* have been identified based on molecular docking method and this shows the potential of these plant-derived chemicals to bind with AeSCP-2 and inhibit its action [5]. Furthermore, the same technique revealed that the terpene, *α*-amyrin extracted from *Calotropis gigantea*, has an effective AeSCP-2 inhibitory potential [50].

Synthetic analogs of curcumin are very easy to synthesize and a very rich source of potential insecticides due to diverse chemical structure. But still the synthesis of these compounds requires advance laboratory settings, and the cost of the process is also high. Considering the requirement of a less expensive and locally available material for vector control, phytochemicals could be selected over synthetic compounds.

However, very few compounds with AeSCP-2 inhibitory activity are discovered so far. Among the few compounds, *α*-mangostin is shown to be more promising against the control of *Ae. aegypti* with the lowest 24 h LC_50_ values (Table 2). However, more compounds will be discovered in the future, and the effectiveness of these compounds on other species should be assessed before selecting the best compound to be used against insect vectors.

## 4. Challenges and Future Perspectives

Insecticide resistance is one of the main issues in the control of vector-borne diseases. The first report for insecticide resistance was reported in the 1940s with the excessive use of DDT [8]. At least, 100 species of mosquitoes and other disease-transmitting vectors are reportedly resistant to insecticides [51]. There is always a chance of developing resistance in insects against insecticides despite the nature of the insecticide, whether it is a biological control agent or a chemical compound [52, 53]. Therefore, any reason for the development of such resistance must be prevented. The resistance can be managed through removing selection pressure by rotation of insecticides or killing resistant strains by using a mixture of insecticides [54]. Use of integrated vector control approaches is also recommended to minimize the risk of developing resistance and maximize the effectiveness of vector control [55].

Furthermore, it is vital to make sure that the insecticides have no effect on other animals. Most of the compounds tested in reported studies are specific for mosquito SCP-2, indicating a less likelihood of harming nontargeted species. However, further studies are necessary to confirm the environmental friendliness.

It is also important to pay attention to the practical considerations associated with the use of these compounds in the control of disease-transmitting vectors. For instance, the persistence of the compound in the environment must be determined and precautions must be taken to maximize the half-life of these compounds in the environment. However, the time must be enough to be effective on the vectors and should not be too persistent to have other environmental impacts. The use of naturally occurring compounds such as *α*-mangostin may exclude this problem as it does not cause other environmental effects [56]. *α*-Mangostin was found to be having reduced larvicidal activity upon exposure to sunlight for 6 h [45]. This is the issue associated with most of the phytochemicals as they are not photostable. Therefore, these compounds will require careful preparation such as slow release formulations combined with UV-blocking additives as used for methoprene larvicides which allowed them to remain biologically active for months in the outside environment [45, 57]. Furthermore, the plant species explained here may not be available in all geographical settings. And the discovery of more and more compounds is essential for the mixed use of insecticides.

A large range of other plant extracts with a potential AeSCP-2 inhibitory activity has shown insecticidal effects against various species of mosquitoes [58–61]. However, the exact mode of action of these compounds is yet to be understood. Therefore, studies should be focused on confirming the mode of action of these compounds as the inhibition of AeSCP-2 acts more specifically on a target group of insects in relation to compounds with a different mode of actions.

Interestingly, SCP-2 is not critical for survival in vertebrates [62, 63]. Moreover, empirical evidence has confirmed that *α*-mangostin has no adverse biological impact on rats [45]. Therefore, it is apparent that the compounds which inhibit cholesterol uptake in insects are less likely to be harmful to mammals. As shown by Kim et al. [42], *α*-mangostin also affects *Manduca sexta*, which is a species of the order Lepidoptera, a totally different order from mosquitoes, which means these compounds may also act on insects other than medically important species. Therefore, these compounds must be used with care preventing the exposure of other beneficial insects to them. Molecular level studies have revealed the structural differences that cause the differential specificity of SCP-2 inhibitory compounds [39, 64]. These studies and future studies on the structural characteristics of SCP-2 molecules of other species may provide insights into how we can make structural alterations to SCP inhibitor molecules in the way of increasing the specificity, targeting only one group or a single species of insects. However, this requires a lot of long-term molecular level studies and high-cost laboratory procedures and thus may take a long time to achieve. Almost no studies are conducted on the possibility of using this method against medically important insects other than mosquitoes. Therefore, exposure trials are required to assess the effectiveness of these SCP inhibitors against other insect vectors, especially, sand flies, kissing bugs, and tsetse flies, which are responsible for a large number of deaths in African and Asian regions, annually.

## 5. Conclusions

Inhibition of sterol carrier proteins (SCPs) in mosquitoes and other insect vectors using a compound that acts through competitive binding is a promising method in controlling mosquito vector populations in disease-endemic areas if used with proper care and integrated approaches. Currently, available knowledge is limited and more studies are required to fill the gaps of understandings.

## Figures and Tables

**Table 1:** Synthetic organic compounds with AeSCP-2 inhibitory activity against *Ae. aegypti* [42, 43].

Compounds	(IC_50_/EC_50_) *µ*M
*SCPIs*	
*N*-(4-{[4-(3,4-Dichlorophenyl)-1,3-thiazol-2-yl]amino}phenyl)acetamidehydrobromide **(SCPI-1)**	0.347 (IC_50_)
8-Chloro-2-(3-methoxyphenyl)-4,4-dimethyl-4,5-dihydroisothiazolo[5,4-c]quinoline-1(2H)-thione **(SCPI-2)**	0.059 (IC_50_)
3-(4-Bromophenyl)-5-methoxy-7-nitro-4H-1,2,4-benzoxadiazine **(SCPI-3)**	0.159 (IC_50_)
4,4,8-Trimethyl-5-(3-methylbutanoyl)-4,5-dihydro-1H-[1,2]dithiolo[3,4-c]quinoline-1-thione **(SCPI-4)**	0.065 (IC_50_)
3-Bromo-N-{2-[(4-chloro-2-nitrophenyl)amino]ethyl}-4-ethoxybenzamide **(SCPI-5)**	0.042 (IC_50_)

*Synthetic analogs of curcumin*	
(2E,6E)-2-6-Bis(3,4,5-trimethoxybenzylidene) cyclohexanone	12.11 (EC_50_)
(2E,6E)-2-6-Bis(furan-2-ylmethylene) cyclohexanone	62.87 (EC_50_)
(1E,4E)-1,5-Bis(4-hydroxyphenyl) penta-1,4-dien-3-one	2.38 (EC_50_)
(1E,4E)-1-(2-Hydroxyphenyl)-5-phenylpenta-1,4-dien-3-one	2.02 (EC_50_)
1,5-Bis-(3,4,5-trimethoxy-phenyl)-penta-1,4-dien-3-one	0.65 (EC_50_)

**Table 2:** Phytochemicals with SCP-2 inhibitory activity against medically important insects.

Compound/extraction	Plant species	Part of the plant	LC_50_ *μ*gml^−1^	Reference
*α*-Mangostin	*Garcinia mangostana*	Pericarp of the fruit	19.4 *μ*gml^−1^ (*Ae. aegypti*)	Ee et al. [44]
			5.0 (*Ae. aegypti*)	Larson et al. [45]
			4.0 (*An. gambiae*)	
			11.0 (*An. quadrimaculatus*)	
			6.0 (*An. stephensi*)	
			6.0 (*Cx. pipiens pipiens*)	
			8.0 (*Cx. pipiens quinquefasciatus*)	
			23.3 (*Ae. aegypti*)	Sasikumar and Ghosh, [49]
Quercetin	*Saxifraga stolonifera*	Whole plant	7.53 *μ*gml^−1^ (*Ae. aegypti*)	Anstrom et al. [43]
Dulxanthone	*Garcinia mangostana*	Pericarp of the fruit	8.81(*Ae. aegypti*)	Sasikumar and Ghosh, [49]

## Abbreviations

SCP-2: Sterol carrier protein-2
DDT: Dichlorodiphenyltrichloroethane
JH: Juvenile hormone
SCPIs: Sterol carrier protein inhibitors.
